# Long Non-coding RNAs as Important Biomarkers in Laryngeal Cancer and Other Head and Neck Tumours

**DOI:** 10.3390/ijms20143444

**Published:** 2019-07-12

**Authors:** Alessia Maria Cossu, Laura Mosca, Silvia Zappavigna, Gabriella Misso, Marco Bocchetti, Federica De Micco, Lucio Quagliuolo, Marina Porcelli, Michele Caraglia, Mariarosaria Boccellino

**Affiliations:** 1 Biogem scarl, Institute of Genetic Research, Laboratory of Molecular and Precision Oncology, 83031 Ariano Irpino (AV), Italy; 2 Department of Precision Medicine, University of Campania “Luigi Vanvitelli”, 80100 Naples, Italy

**Keywords:** head and neck cancer (HNC), head and neck squamous-cell carcinoma (HNSCC), laryngeal squamous cell carcinoma (LSCC), laryngeal cancer (LC), long ncRNA (lncRNA), prognostic biomarker

## Abstract

Head and neck carcinoma (HNC) is a heterogeneous disease encompassing a variety of tumors according to the origin. Laryngeal cancer (LC) represents one of the most frequent tumors in the head and neck region. Despite clinical studies and advance in treatment, satisfactory curative strategy has not yet been reached. Therefore, there is an urgent need for the identification of specific molecular signatures that better predict the clinical outcomes and markers that serve as suitable therapeutic targets. Long non-coding RNAs (lncRNA) are reported as important regulators of gene expression and represent an innovative pharmacological application as molecular biomarkers in cancer. The purpose of this review is to discuss the most relevant epigenetic and histological prognostic biomarkers in HNC, with particular focus on LC. We summarize the emerging roles of long non-coding RNAs in HNC and LC development and their possible use in early diagnosis.

## 1. Introduction

Head and neck cancer (HNC) is a type of malignant tumor that occurs in the upper neck, like the tongue, gingiva, nasopharynx, larynx, and thyroid [1,2]. The initiation and progression of HNC is a rather complex multistep process involving genetic and epigenetic changes. The most common histological subtype of HNC is referred to head and neck squamous cell carcinoma (HNSCC) [3] and laryngeal squamous cell carcinoma (LSCC) [4,5].Tobacco smoking, alcohol consumption and human papilloma virus (HPV) infections are the most common risk factors. Recently, the five-year overall survival rate for HNSCC patients has remained at about 50%, despite the progress made in overall therapy [6,7]. Many HNCs present metastasis at the time of diagnosis and there are currently no tools available to monitor HNSCC patients for early stages of local recurrences or distant metastases. In this light, there is a strong interest to identify novel biomarkers that can select patients that would benefit from a given therapy, and can predict the clinical outcome. In this context, multiple types of RNA, including long non-coding RNAs (lncRNAs), circular RNAs, and microRNAs (miRNAs) regulate the expression levels of various mRNAs in mammalian cells [8]. Recent evidences have highlighted the importance of lncRNAs as critical regulators of gene expression and tumorigenesis [9]. LncRNAs are a heterogenous group of non-coding polyadenylated RNAs longer than 200 nucleotides [10]. Similar to miRNAs, lncRNAs are closely associated with multiple human diseases, including cancer [11]. LncRNAs are involved in different physiological and pathological aspects through different mechanisms of action [12]. Moreover, lncRNAs seem to be implicated in cancer progression, such as tumor development and metastases. LncRNAs stably circulate in human body fluids and can be obtained with non-invasive methods, representing a potential prognostic factor for HNCs. Since lncRNAs can be easily detected in tissues and serum, they have been suggested to be promising candidates as diagnostic and prognostic tumor biomarkers and for development of novel therapeutic approaches. [11]. Here, we classify lncRNAs according to different HNC tumor types with particular emphasis on LSCC, and the effects of tumor-associated lncRNAs and their corresponding downstream molecules on the tumor are also described.

## 2. Biological Knowledge of Laryngeal and Head and Neck Cancers

HNCs are the sixth most frequent cancer worldwide [1,2]. LCs represent one-third of all HNCs, occurring in different subsites of larynx, with different symptoms and treatments. Approximately 300,000 deaths occur each year and over 500,000 new cases are diagnosed [13]. Squamous cell carcinoma (SCC) is the major pathological type and more than 40% of patients are characterized by an advanced stage of tumor. Head and neck squamous carcinoma (HNSCCs) are epithelial tumors showing evidence of squamous differentiation, characterized by aggressive and invasive phenotypes that affect multiple surrounding tissues: nasal cavity, mouth, salivary glands, larynx, pharynx and metastasize to distant organs, even at early stages [10]. HNSCC arises from multiple anatomic locations in the head and neck region. In the context of HNCs, oral squamous cell carcinoma (OSCC) is the fourth most common oral [11,12] cancer, with high incidence in men compared to women. Nasopharyngeal carcinoma (NPC) is more frequent in specific geographical areas, such as North Africa, Southeast Asia and Southern China [13]. NPC includes two types—differentiated and undifferentiated carcinoma. Early diagnosis of NPC is difficult due to unobvious symptoms and patients are mostly diagnosed in advance and/or metastatic stages [14].Thyroid cancer (TC) is classified into three histological types according to tumor origin, in particular, papillary thyroid cancer (PTC), follicular thyroid cancer (FTC) and anaplastic thyroid cancer (ATC) deriving from follicular thyroid cells [15]. Esophageal squamous cell carcinoma (ESCC) is the most prevalent and aggressive type of esophageal cancers, determined by both environmental and genetic factors [16].The diagnosis of HNSCC and LSCC are made according to the World Health Organization (WHO) classification from a surgical biopsy sample. Alcohol, tobacco consumption and infection are causative factors for HNSCC and LSCC. Human papilloma virus (HPV) is considered as an independent risk factor [17]. HPV tumor positivity can lead to mutations in the PIK3CA gene, loss of the TRAF3 gene, and amplification of the E2F1 gene. On the other hand, tumors that are negative for HPV can have mutations in CCND1, FADD, BIRC2, YAP1, CASP8 or HRAS and in other genes involved in the regulation of cell cycle, cell death and NF-kB. Mutations of TP53 are associated with tobacco consumption, inactivation of CDKN2A and changes in genes associated with oxidative stress. A recent evidence has reported the presence of different mutations in PIK3CA, CASP8, NOTCH1 or TP53 genes in oral tumors with better treatment response and mutations in NSD1, WNT and NFE2L2 genes in LC [18].Molecular profiling of genes that influence HNSCC and LC proteins is very important, as it represents a valid tool for testing selected biomarkers. Many factors positively involved in the expression of the diseases are considered as predictive or prognostic factors [19]. LncRNAs can be used as new and promising classes of biomarkers also because they are often dysregulated in HNCs [20].

## 3. Classification and Characteristics of Non-coding RNAs

**Figure 1:**
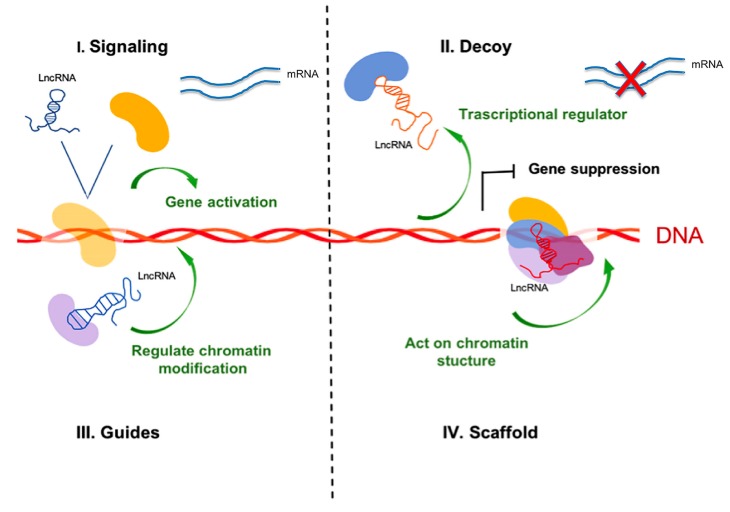
Classification of lncRNA based on the mechanism of action. LncRNA-mediated transcriptiona lregulation through four archetypes based on their molecular mechanism. LncRNAs that belong to the “signaling archetype”: they act as molecular signal activating or silencing genes depending on the stimulus (**I**); “decoy archetype”: the lncRNAs act asdecoys that bind to and interfere with another sequence or structure for binding, considered like negative regulators (**II**); “guide archetype”: the lncRNAs bind to specific proteins and transport them to the specific target sequence. The interaction may be direct (between lncRNA-protein complex and the DNA) or indirect (between lncRNA-protein and protein-DNA complexes) (**III**); “scaffold archetype”: lncRNAs act by bringing proteins into a complex or in spatial proximity to facilitate the interaction (**IV**).

**Figure 2:**
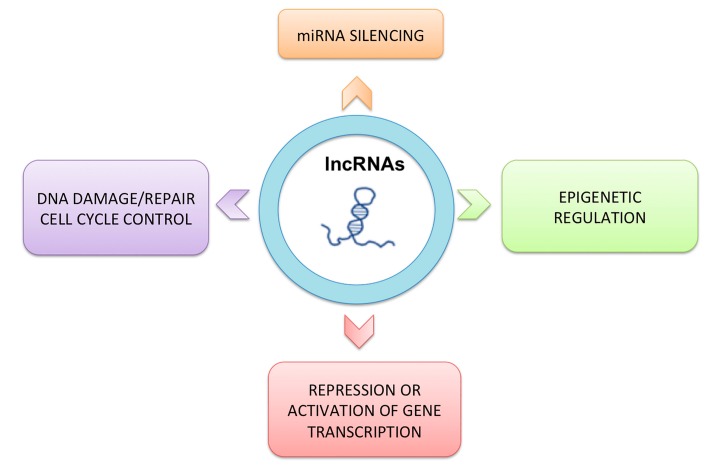
Function of long non-coding RNAs. LncRNAs play important roles in different biological processes.

Non-coding RNAs (ncRNAs), represent novel regulatory layers in the transcriptional and post-transcriptional gene regulation. They have functional roles in various aspects of gene regulation, such as epigenetic regulation, X chromosome inactivation, genomic imprinting, nuclear and cytoplasmic trafficking, transcription, and mRNA splicing [21]. ncRNAs can be divided into two classes: housekeeping ncRNAs and regulatory ncRNAs. The first class includes transfer RNAs (tRNA), ribosomal RNAs (rRNA), small nuclear RNAs (snRNA) and small nucleolar RNAs (snoRNA) and are usually expressed constitutively and required for normal function and viability of the cell. The second class includes two subgroups: (i) short ncRNAs like microRNAs (miRNA), small interfering RNAs (siRNA) and Piwi-interacting RNAs (piRNAs) and (ii) long ncRNAs such as antisense lncRNAs (aslncRNA) and enhancer RNAs (eRNA)—these are expressed at certain stages of development, during cell differentiation, or as a response to external stimuli, which can affect the expression of other genes. Abnormal miRNA expression determines a wide variety of cellular pathways governing human malignancies, such as cell proliferation, apoptosis, metastasis, and drug response [22,23,24]. LncRNAs are larger than 200 nucleotides and are the most transcribed sequences, yet not protein-coding; they may be located in cytosolic or nuclear fractions. The lncRNA biogenesis is mediated by RNA polymerase II similarly to that of mRNA (Figure 1). LncRNAs can be polyadenylated, appear with alternative cleavage, alternative polyadenylation, and alternative splicing, leading to different isoforms from the same locus, and are often transcribed from either strand within a protein-coding locus [10,11]. Several evidences indicate that lncRNAs play an important role in various biological processes such as gene expression, chromatin modification, occurrence and progression of cancers (Figure 2). Exosomal lncRNAs can be detected in body fluids and their deregulation is associated with tumor growth, invasion, angiogenesis, metastases and chemo-resistance. Thus, the functional meaning of exosome-derived lncRNAs in cancer biology suggests the possibility of using them as promising, non-invasive biomarkers for cancer prognosis and therapy [25,26,27].

Specifically, functional domains of lncRNAs’ secondary or tertiary structure can facilitate interaction with chromatin, RNA and proteins; therefore, lncRNAs can have a transcriptional and post-transcriptional control through chromatin remodeling in HNC metastases [28], activate or silence gene expression [29], regulate gene transcription, and process mRNA/miRNA. In a recent study on colon cancer (CRC), lncRNA transcript 2 (CCAT2) was elevated in CRC stem cells and was capable to repress miR-145, involved in proliferation and differentiation. Recent evidences have shown a new feedback loop involving MALAT-1 as miRNA sponge capable of attenuating miR-200c-3p function in pancreatic ductal adenocarcinoma (PDAC) [30]. Moreover, a lncRNA highly up-regulated in liver cancer (HULC) may act as an endogenous ‘sponge’, which down-regulates a series of miRNA activities, including miR-372 [31]. Cytoplasmic lncRNAs may modulate gene expression, improve or attenuate miRNA stability and actas miRNA sponges [29,32].

## 4. Prognosis Associated lncRNAs in HNC

An increasing number of lncRNAs has been found to play roles in HNC initiation and progression, suggesting that they could function as novel biomarkers and therapeutic targets to provide more effective diagnosis, prognosis and treatment for HNC patients [7]. Therefore, we tried to focus our attention on widely studied lncRNAs and some recently identified lncRNAs, classifying them according to the different tumor types (ESCC, HNSCC, NPC, OSCC, PTC and TSCC) and highlighting their pathogenetic role in HNC.

### 4.1. HOTAIR

The long non-coding HOX transcript antisense RNA (HOTAIR) with 2158-nucleotidesis located at chromosome 12q13.13. It plays significant roles in promoting cancer metastasis. It is an epigenetic regulator that plays a central role in the initiation and progression of many types of human tumors, such as CRC, ESCC, and PDAC. The expression of HOTAIR is related to oncogenesis, metastasis and poor prognosis in HNC [33,34,35]. In fact, the expression level in tumor tissues was significantly higher than that in paracancerous tissues and correlated with tumor size and clinical stage. The suppression of HOTAIR expression in Tca8113 cells significantly retarded cell growth, arrested cell cycle, and promoted apoptosis [35]. In NPC tumor tissues, HOTAIR overexpression promotes angiogenesis and tumor growth by directly activating the transcription of vascular endothelial growth factor-A (VEGF-A) and glucose-regulated protein 78 (GRP78) as well as through GRP78-mediated upregulation of VEGFA and Ang2 expression [36]. In fact, HOTAIR knockdown significantly inhibited both in vitro and in vivo tumor cell growth and angiogenesis [36] (Table 1).

### 4.2. UCA1

LncRNA urothelial cancer-associated 1 (UCA1) is overexpressed in TSCC tumor tissues compared to neighboring non-tumor tissues. It is involved in cancer invasion and metastasis of TSCC. Indeed, patients with lymph node metastasis express much higher levels of UCA1 [57]. Over-expression of UCA1 lncRNA could promote metastatic but not proliferation ability of TSCC cells (Table 1).

### 4.3. FOXC1

FOXC1 upstream transcript (FOXCUT) is alncRNA that is overexpressed in OSCC and promotes tumor cell proliferation and migration by regulating FOXC1 expression. The down-regulation of FOXCUT by small interfering RNA (siRNA) decreased the expression of FOXC1. Moreover, in OSCC cells, Tca8113 and SCC-9, down-regulation of either FOXC1 or FOXCUT by siRNA inhibited cell proliferation and migration in vitro with a concomitant reduction of MMP2, MMP7, MMP9, and VEGF-A. In conclusion, FOXC1 may be co-amplified with FOXCUT in OSCC, and both of them may be functionally involved in the tumor progression of OSCC. FOXC1 mRNA is positively associated with FOXCUT transcript. Indeed, FOXCUT silencing reduces FOXC1 expression both at mRNA and protein level [45] (Table 1).

### 4.4. AFAP1-AS1

The lncRNA actin filament-associated protein 1 antisense RNA1 (AFAP1-AS1) is overexpressed in NPC and is related to its progression and poor survival. In vitro experiments demonstrated that AFAP1-AS1 knockdown significantly inhibited NPC cell migration and invasion. AFAP1-AS1 knockdown also increased AFAP1 protein expression. Proteomic and bioinformatics analyses suggested that AFAP1-AS1 affected the expression of several small GTPase family members and molecules in the actin cytoskeleton signaling pathways. AFAP1-AS1 promoted cancer cell metastasis via regulation of actin filament integrity through small Rho/Rac GTPase signaling pathways. In nude mice, AFAP1-AS1 knockdown represses cell invasion and inhibits NPC lung metastasis [38] (Table 1).

### 4.5. HNF1A-AS

The lncRNA hepatocyte nuclear factor 1A-antisense RNA (HNF1A-AS) is up-regulated in NPC tissues increasing cell cycle progression, tumor cell proliferation, and migration. Lentivirus-mediated HNF1A-AS knockdown suppressed cell proliferation and migration abilities of NPC cells. In mice injected with CNE-2 and SUNE-1, depletion of HNF1A-AS caused inhibition of tumor growth, whereas cell cycle analysis showed that HNF1A-AS-knockdown resulted in cell accumulation in the G0/G1 phase. Moreover, HNF1A-AS was found to be associated with epithelial to mesenchymal transition. In particular, HNF1A-AS increased the levels of the mesenchymal proteins N-cadherin and vimentin and reduced the level of epithelial protein E-cadherin [48] (Table 1).

### 4.6. ROR

LncRNA reprogramming (lncRNA-ROR) is related to NPC chemo-resistance. Its expression is higher in NPC tissues compared with the normal and is positively correlated with cell proliferation, metastasis and suppression of apoptosis. In fact, inhibition of the expression of ROR increased G1 phase (from 59.91% to 70.11%), and decreased the S and G2 phase (from 40.01% to 29.89%) in NPC CNE2 cells. In addition, overexpression of lncRNA-ROR induced chemo-resistance to cis-platin in NPC cells. Several data indicate that translational p53 and p21 regulation after chemotherapy may play an important role in chemo-resistance. The authors have explored the relationship between lncRNA-ROR and p53. CNE2 cells were first transfected with negative control or siRNA against ROR and then treated with DDP at 2 μg/mL for 24 h before harvesting for western blot. Notably, knockdown of ROR leads to an increase in the activation of p53 pathways [50] (Table 1.).

### 4.7. LET

The lncRNA-Low Expression in Tumor (lncRNA-LET) is down-regulated in NPC tissues and cell lines. Decreased LET expression significantly correlated to advanced clinical stage, larger tumor size, increased lymph node tumor burden, and poor survival of NPC patients. Gain- and loss-of-function experiments demonstrated that enhanced LET expression inhibited NPC cells proliferation and induced apoptosis. By contrast, the knockdown of LET promoted NPC cells proliferation and inhibited apoptosis. Moreover, lncRNA-LET is transcriptionally repressed by EZH2-mediated H3K27 histone methylation on the LET promoter. The expressions of EZH2 and lncRNA-LET are significantly inversely correlated in NPC tissues [49]. Therefore, LET can be considered a target of EZH2 oncogenic function in NPC (Table 1).

### 4.8. PlncRNA-1

PlncRNA-1 prostate cancer-up-regulated long noncoding RNA 1, (also known as CBR3-AS1), located in the antisense region of carbonyl reductase 3 (CBR3), is overexpressed in cell lines and tissues of prostate cancer (CaP). Knockdown of PlncRNA-1 expression in CaP cells leads to a decrease in cell proliferation and an increase in apoptosis by regulating the androgen receptor. In esophageal squamous cell carcinoma (ESCC), the expression of PlncRNA-1 was significantly higher in human ESCC compared with the adjacent noncancerous tissues (69.8%, *p* < 0.05), and this observation correlated with advanced clinical stage (*p* < 0.01) and lymph node metastasis (*p* < 0.05). Furthermore, knockdown of PlncRNA-1 reduced cell proliferation and increased the apoptosis in vitro [39,40,41] (Table 1).

### 4.9. GAS8-AS1

Recently, a study has identified somatic mutations for Chinese PTC using 402 tumor-normal pairs (screening: 91 pairs via exome sequencing; validation: 311 pairs via Sanger sequencing). Three distinct mutational signatures were found different from the two signatures reported in Caucasian PTC. Ten significantly mutated genes were identified. Notably, lncRNA GAS8-AS1 is the secondary most frequently altered gene and acts as a novel tumor suppressor in PTC. As mutation hotspot, the c.713A>G/714T>C dinucleotide substitution was found among 89.1% patients with GAS8-AS1 mutations and associated with advanced PTC disease (*p* = 0.009). Interestingly, the wild-type lncRNA GAS8-AS1 (A713T714) showed consistently higher capability to inhibit cancer cell growth compared to the mutated lncRNA (G713C714). Further studies also elucidated the oncogene nature of the G protein-coupled receptor LPAR4 and its c.872T>G (p.Ile291Ser) mutation in PTC malignant transformation. Together, this study defines an exome mutational spectrum of PTC in the Chinese population and highlights lncRNA GAS8-AS1 and LPAR4 as potential diagnostic and therapeutic targets. Furthermore, GAS8-AS1 mutations in PTC are associated with an advanced clinical stage [46] (Table 1).

### 4.10. ADAMTS9-AS2

LncRNA ADAMTS9-AS2 is the most significantly upregulated in TSCC tissues from patients with lymph node metastasis and is associated with poor prognosis. Furthermore, ADAMTS9-AS2 knockdown in TSCC cells leads to the inhibition of cell migration and invasion and reverses TGF-β1 induced EMT. ADAMTS9-AS2 knockdown also inhibits TSCC cell growth in vitro and in vivo. In addition, ADAMTS9-AS2 is a cytoplasmic lncRNA that shares the miRNA response elements (MREs) of miR-600 with EZH2, as confirmed by a luciferase reporter assay and AGO2-dependent RNA immunoprecipitation (RIP). These results demonstrate an explicit oncogenic role of ADAMTS9-AS2 in TSCC tumorigenesis via competition with miR-600, suggesting a new regulatory mechanism of ADAMTS9-AS2 and providing a potential therapeutic target for TSCC patients [37] (Table 1).

### 4.11. ESCCAL-1

In a recent study, alncRNA array was used for coding and non-coding RNA expression. R program and Bioconductor packages (limma and RedeR) were used for differential expression and co-expression network analysis, followed by independent confirmation and functional studies of inferred onco-lncRNA ESCCAL-1 using quantitative real time polymerase chain reaction, small interfering RNA-mediated knockdown, apoptosis and invasion assays in vitro. The global coding and lncRNA gene expression pattern is able to distinguish ESCC from adjacent normal tissue. The co-expression network from differentially expressed coding and lncRNA genes in ESCC was constructed, and the lncRNA function may be inferred from the co-expression network. LncRNA ESCCAL-1 is a predicted novel onco-lncRNA, and is overexpressed in 65% of an independent ESCC patient cohort (*n* = 26). Moreover, knockdown of ESCCAL-1 expression increases esophageal cancer cell apoptosis and reduces the invasion in vitro [42] (Table 1).

### 4.12. PVT1

In a recent study, a total of 84 patients who were diagnosed as having thyroid cancer (papillary thyroid carcinoma (PTC), follicular thyroid carcinoma (FTC), and anaplastic thyroid carcinoma (ATC)) were enrolled. Expression of lncRNA PVT1 in thyroid cancer tissues and cell lines (IHH-4, FTC-133, and 8505C) was analyzed using RT-polymerase chain reaction (PCR) and western blotting analysis. The effects of lncRNA PVT1 expression on thyroid cancer cell proliferation and cell cycle were analyzed using flow cytometry. Furthermore, the effects of lncRNA expression on thyroid-stimulating hormone receptor (TSHR) expression and polycomb enhancer of zeste homolog 2 (EZH2) were also analyzed using RNA immunoprecipitation (RIP) assay and chromatin immunoprecipitation (ChIP) assay, respectively. Compared to the controls, lncRNA PVT1 was significantly up-regulated in thyroid tissues, as well as in the three tumor cell lines (*p* < 0.05). Silenced PVT1 significantly inhibited thyroid IHH-4, FTC-133, and 8505C cell proliferation and arrested cell cycle at G0/G1 stage and significantly decreased cyclin D1 and TSHR expression (*p* < 0.05). Moreover, lncRNA PVT1 could be enriched by EZH2, and silencing PVT1 resulted in the decreased recruitment of EZH2 [56]. This study suggested that lncRNA PVT1 may contribute to tumorigenesis of thyroid cancer through recruiting EZH2 and regulating TSHR expression (Table 1).

### 4.13. PTCSC2- PTCSC3

He et al. recently found a novel long intergenic noncoding RNA gene and named it papillary thyroid cancer susceptibility candidate 2 (PTCSC2). Transcripts of PTCSC2 are down-regulated in PTC tumors. The risk allele [A] of rs965513 was significantly associated with low expression of unspliced PTCSC2, FOXE1, and TSHR in unaffected thyroid tissue. PTCSC2 unspliced transcript levels were associated with age and CLT. The correlation between the rs965513 genotype and the PTCSC2 unspliced transcript levels remained significant after adjusting for age, gender, and CLT. Forced expression of PTCSC2 in the BCPAP cell line affected the expression of a subset of noncoding and coding transcripts with enrichment of genes functionally involved in cell cycle and cancer [54]. Similarly, a unique, long, intergenic, noncoding RNA gene (lincRNA) named Papillary Thyroid Carcinoma Susceptibility Candidate 3 (PTCSC3) located 3.2 kb downstream of rs944289 at 14q.13.3 and its expression is strictly thyroid specific. By quantitative PCR, PTCSC3 expression was strongly down-regulated in thyroid tumor tissues of 46 PTC patients and the risk allele (T) was associated with the strongest suppression. The SNP rs944289 was located in a binding site for the CCAAT/enhancer binding proteins (C/EBP) α and β. The risk allele destroyed the binding site in silico. Both C/EBPα and C/EBPβ activated the PTCSC3 promoter in reporter assays (*p* = 0.0009 and *p* = 0.0014, respectively) and the risk allele reduced the activation compared with the non-risk allele (C) (*p* = 0.026 and *p* = 0.048, respectively). Restoration of PTCSC3 expression in PTC cell lines (TPC-1 and BCPAP) inhibited cell growth (*p* = 0.002 and *p* = 0.019, respectively) and affected the expression of genes involved in DNA replication, recombination and repair, cell movement, tumor morphology, and cell death [55] (Table 1).

### 4.14. FIRRE

LncRNA FIRRE (Functional Intergenic Repeating RNA Element) interacts with the nuclear matrix factor hnRNPU and its localization occurs through at least five distinct loci on the X chromosome. Knockdown of hnRNPU or deletion of Firre locus results in a loss of nuclear organization. [43]. Nohata et al., using TCGA HNSCC RNA-sequencing data from 426 HNSCC and 42 adjacent normal tissues, found 728 lncRNA transcripts significantly and differentially expressed in HNSCC. Among the 728 lncRNAs, 55 lncRNAs were significantly associated with poor prognosis, such as overall survival and/or disease-free survival. Next, 140 lncRNA transcripts significantly and differentially expressed between HPV positive and negative tumors. Thirty lncRNA transcripts were differentially expressed between TP53 mutated and TP53 wild type tumors. Among the thirty aberrantly expressed transcripts, FIRRE was found to be up-modulated in both tumor samples and HNSCC cell lines. Recently, FIRRE was reported to interact with the nuclear matrix factor hnRNPU. FIRRE localizes across at least three distinct trans-chromosomal loci. Both genomic excision of FIRRE locus and knockdown of hnRNPU shows decreased co-localization of these trans-chromosomal interacting loci. Thus, aberrant epigenetic alterations caused by deregulated FIRRE levels may represent a novel HNSCC oncogenic mechanism, which should be clarified in future experimental studies [44] (Table 1).

### 4.15. H19

Guan et al. recently reported that both H19 and miR-675 were significantly overexpressed in a cohort of 65 primary tumor samples and two HNSCC cell lines. Increased expression of either H19 or miR-675 was significantly correlated with higher risk of patient relapse, and associated with worse overall survival and poor disease-free survival. The reduction of miR-675 expression decreased cell viability, migration and invasion. Taken together, these results suggest that the strong correlation of H19 overexpression together with higher miR-675 and lymph node metastases could be useful predictive markers [56]. The long non-coding RNA (lncRNA) H19 is overexpressed also in NPC tissues; knockdown of H19 significantly inhibited the invasive ability of NPC cells. Moreover, H19 affected the expression of EZH2, which has also been observed to be up-regulated in NPC and to promote cell invasion. Rather than direct interaction, H19 decreased EZH2 expression by suppressing the activity of miR-630, which is a repressor of EZH2 and interacts with H19 in a sequence-specific manner. Furthermore, H19 inhibited E-cadherin expression and promoted cell invasion of NPC cells via the miR-630/EZH2 pathway [47] (Table 1).

### 4.16. MEG3

LncRNAs Maternally Expressed Gene 3 (MEG3), a tumor suppressor, is not expressed in TSCC. The loss of MEG3 expression promotes tumor progression but its re-expression inhibits proliferation and induces apoptosis. Low MEG3 levels are associated with high TSCC mortality and poor overall survival. MEG3 overexpression inhibits tumorigenesis by increasing the expression of p53 and Growth Differentiation Factor 15 (GDF15). MEG3 increases p53 levels by reducing its ubiquitin-proteasome-mediated degradation. The reduction of the miR-26a levels in TSCC down-regulates MEG3 by increasing the DNA methylation levels [53] (Table 1).

### 4.17. MALAT-1

MALAT-1 lncRNA levels were increased in TSCCs, especially in those with lymph node metastasis (LNM). Knockdown of MALAT-1 lncRNA in TSCC cells led to impaired migration and proliferation ability in-vitro and fewer metastases in-vivo. DNA microarray analysis showed that several members of small proline rich proteins (SPRR) were up-regulated by knockdown of MALAT-1 in TSCC cells. SPRR2A over-expression could impair distant metastasis of TSCC cells in-vivo. Consequently, knockdown of MALAT-1 lncRNA in TSCC cells led to impaired migration and proliferation in vitro and less metastases in vivo [52] (Table 1).

### 4.18. LOC541471

Wu et al. performed a RNA-Seq dataset containing 43 tumor-normal pairs. An independent t-test identified that the expression level of lncRNA LOC541471 was significantly increased in tumor tissues compared to healthy tissues. Additionally, high lncRNA LOC541471 expression was significantly associated to increasing lymph node metastasis and perineural invasion. A multivariate Cox regression analysis revealed that high lncRNA LOC541471 expression was an independent predictor for reduced overall survival (*n* = 487) and relapse-free survival (*n* = 355). According to the anatomic derivation of the neoplasms, HNSCC samples were classified as oropharyngeal carcinoma (*n* = 297), oral carcinoma (*n* = 80), laryngeal carcinoma and hypopharyngeal carcinoma (*n* = 123). A negative association was revealed between lncRNA LOC541471 expression and overall survival in all subtypes of HNSCC. Since IncRNA LOC541471 is associated with poor overall survival in HNSCC patients, it can be considered a potential prognostic factor [51] (Table 1).

## 5. LncRNAs in Laryngeal Cancer

### 5.1. DGCR5

The two human LSCC lines (Hep-2 and Hep-2R) have different radio-sensitivities when cultured. Interestingly, CSC-like phenotypes were much more enriched in Hep-2R cells. In human larynx squamous carcinoma cell lines, Hep-2R, lncRNA DiGeorge syndrome critical region gene 5 (DGCR5) is upregulated and microRNA-506 is down-regulated. In addition, silencing of DGCR5 inhibited the stemness and enhanced the radiosensitivity of Hep-2R cells. On the other hand, overexpression of miR-506 also suppressed the CSC-like traits and the radio-sensitivity was significantly increased. In addition, miR-506 was predicted as target of DGCR5 and the correlation between them was validated. Finally, DGCR5 inhibition could repress Wnt signaling, that exerted a significant role in human laryngeal CSCs activity, by sponging miR-506. In in vivo assays, DCGR5 depressed stemness of human LC cells through modulating miR-506 and Wnt signaling pathway. The overexpression of lncRNA DGCR5 induced cancer stem cell-like properties by sponging miR-506 through activation of Wnt signaling in human laryngeal carcinoma cells [58] (Table 2).

### 5.2. PCAT19

The expression levels of lncRNA PCAT19 are up-regulated in LC tissues and associated with decreased overall survival. Using LC cell lines Hep-2 and AMC-HN-8, it was demonstrated that knockdown of PCAT19 decreased cell proliferation, increased mitochondrial respiration, and inhibited glycolysis. In detail, pyruvate dehydrogenase kinase 4 (PDK4) expression and PDHE1α phosphorylation levels were decreased upon PCAT19 knockdown. Further studies indicated that miR-182 functioned as connection between PCAT19 and PDK4, which could also regulate the cellular metabolism thus affecting cell proliferation. Furthermore, the PCAT19/miR-182/PDK4 axis existed and regulated cell proliferation by modulating glycolysis and mitochondrial respiration. Finally, PCAT19 knockdown decreased the tumor growth in vivo, possibly by regulating the miR-182/PDK4 axis. In conclusion, lncRNA PCAT19 increased cell proliferation and tumorigenesis by modulating the miR-182/PDK4 axis and the metabolism balance. LncRNA PCAT19 might become a promising new target for laryngeal cancer therapy [72] (Table 2).

### 5.3. H19

LncRNA H19 is required for the development and progression of LSCC. In fact, the expression levels of H19 were inversely correlated with the survival rate of LSCC patients. Knockdown of H19 expression inhibited LSCC cell migration, invasion and proliferation. miR-148a-3p was an inhibitory target for H19 and its overexpression reduced LSCC migration, invasion and proliferation, while inhibition of miR-148a-3p did the opposite. The inhibition of LSCC progression induced by H19 knockdown required the activity of miR-148a-3p. DNA methyltransferase enzyme DNMT1 was also a target of miR-148a-3p. Cellular DNA methylation levels were inhibited by both miR-148a-3p overexpression and H19 knockdown. LncRNA H19 promotes LSCC progression via miR-148a-3p and DNA methyltransferase enzyme DNMT [60] (Table 2).

### 5.4. ST7-AS1

LncRNA suppressor of tumorigenicity 7 antisense RNA 1 (ST7-AS1) is an oncogenic factor in LSCC. It is overexpressed in both tissues and cell lines of LSCC and participates in malignancy through migration, tumor sphere formation assay and in vivo implantation. Mechanistically, ST7-AS1 could interact with CARM1, a well characterized protein arginine methyltransferase, and protect CARM1 from ubiquitin-dependent degradation. CARM1 can methylate Sox-2, a pluripotent transcription factor. Thus, ST7-AS1 might mediate its oncogenic effect through CARM1-Sox-2 axis by enhancing Sox-2 self-association and transactivation activity [74] (Table 2).

### 5.5. NEAT1

The lncRNA nuclear paraspeckle assembly transcript1 (NEAT1) is an oncogene in many tumors, including LSCC. In LSCC patients with neck nodal metastasis, NEAT1 is overexpressed. LC cells transduced with NEAT1 siRNA remained in the G1 phase compared to control cells (*p* < 0.05). Flow cytometric analysis showed that the percentage of apoptotic cells was significantly higher in NEAT1 siRNA transduced Hep-2 cells than in cells transduced with GFP lentivirus. To study the effects of NEAT1 inhibition in vivo, 16 mice were subcutaneously injected with Hep-2 cells and all of them developed detectable tumors. The growth of LSCC xenograft was significantly inhibited in mice treated with NEAT1 siRNA lentivirus, compared with mice treated with GFP lentivirus. The average tumor weight in NEAT1 siRNA-treated LSCC xenografts was significantly lower than that in the control group (1.085 ± 0.132 g versus 2.487 ± 0.160 g, *p* < 0.01). The inhibition of the in vivo growth of LC cells was mediated by an increase of cyclin-dependent kinase 6 (CDK6) expression induced by down-regulation of miR-107 [70] (Table 2).

### 5.6. UCA1

A total of 90 patients with LSCC and 90 healthy subjects were enrolled in order to evaluate levels of UCA1 in tumor tissues and adjacent healthy tissues, as well as serum. Receiver operating characteristic curve analysis was performed to evaluate the diagnostic value of serum UCA1 for LSCC. UCA1 levels were significantly higher in tumor tissues compared with adjacent healthy tissues in the majority of patients with LSCC. In addition, serum levels of UCA1 were significantly higher in patients with LSCC compared to healthy controls. UCA1 overexpression promoted, whereas UCA1 knockdown inhibited the proliferation, migration and invasion of LSCC cells. UCA1 overexpression activated the Wnt/β-catenin signaling pathway in LSCC cells, whereas treatment with Wnt inhibitor reduced the enhancing effects of UCA1 overexpression on the proliferation, migration and invasion of LSCC cells. Therefore, UCA1 can promote cell proliferation, invasion and migration of LSCC cells by activating the Wnt/β-catenin signaling pathway [79] (Table 2).

### 5.7. NF90

LncRNA small NF90-associated RNA (snaR) is an oncogenic lncRNA that is up-regulated in plasma of patients with LSCC compared to healthy controls. Plasma levels of snaR increased with stages. Follow-up study showed that high plasma levels of snaR were correlated with poor overall survival. Plasma levels of snaR were positively correlated with transforming growth factor beta (TGF-β1) in patients with LSCC but not in healthy controls. Overexpression of snaR resulted in up-regulation of TGF-β1 in cells of human LSCC cell lines, while exogenous TGF-β1 treatment showed no significant effect on snaR expression. snaR overexpression and exogenous TGF-β1 treatment promoted LSCC cell proliferation, migration, and invasion. In addition, TGF-β inhibitor partially reduced the enhancing effects of snaR overexpression on LSCC cell proliferation, migration, and invasion. Therefore, overexpression of lncRNA snaR is correlated with progression and predicts poor survival of LSCC and the mechanism of its actions is likely related to TGF-β1 [76] (Table 2).

### 5.8. LINC00668

Zhao et al. included in their study the GSE84957 lncRNA expression profile through data mining in the National Center for Biotechnology Information/Gene Expression Omnibus (NCBI/GEO). Then, the differentially expressed genes (DEGs) of LSCC (1646 lncRNAs and 2713 mRNAs, fold change ≥2, *p* ≤ 0.05) were identified from the GSE84957 dataset using bioinformatics analysis. Of the 10 selected differentially expressed lncRNAs, the expression of 7 lncRNAs were verified by qRT-PCR method. LINC00668, a potential carcinogenic lncRNA, was screened out by narrowing down the screening criteria (fold change ≥4, *p* ≤ 0.01). Furthermore, correlation analysis demonstrated that expression levels of LINC00668 were associated with age, pathological differentiation degree, T stage, clinical stage and cervical lymph node metastasis. Moreover, a series of bioinformatics tools and in vitro experiments proved that knockdown of LINC00668 inhibited the proliferation, migration and invasion ability of LSCC cells [65] (Table 2).

### 5.9. SNHG1

Long noncoding RNA (lncRNA) small nucleolar RNA host gene 1 (SNHG1) functions as an oncogene in various human cancers. Gao et al. found that lncRNA SNHG1 was significantly up-regulated in LSCC and associated with prognosis of LSCC patients. Knockdown of SNHG1 inhibited cell proliferation, migration and invasion and induced apoptosis. In addition, knockdown of SNHG1 inhibited LSCC growth and metastasis in vivo. Mechanistically, SNHG1 promotes YAP1 expression and Hippo signaling activity by competitively sponging miR-375. Moreover, YAP1 could occupy the SNHG1 promoter to enhance its transcription, suggesting that a positive loop regulation between YAP1 and SNHG1 exists. Collectively, this study first elucidates the mechanism of SNHG1-mediated malignant phenotypes through evoking the miR-375/YAP1/Hippo signalling axis, which provides a novel target for LSCC treatment [77] (Table 2).

### 5.10. TUG1

Higher lncRNA taurine-up-regulated gene 1 (TUG1) expression in LSCC compared to paired normal tumor-adjacent tissue specimens was observed. Furthermore, high TUG1 expression was positively associated with advanced T category, worse lymph node metastasis and late clinical stage. In vitro experiments with silencing of TUG1 demonstrated that it markedly inhibited viability, invasive and migratory properties of LC Hep-2 cells. Moreover, its suppression caused S-Phase accumulation and G2/M phase decrease and apoptosis. These effects were paralleled by an increase of the expression of p53 that therefore was detected as responsible for these changes [78].

### 5.11. MALAT-1

Zhang et al. used immortalized nasopharyngeal epithelial (NPE) cell line NP-69 as non transformed control and FaDu, Hep-2 and nasopharyngeal carcinoma CNE-2Z cells as transformed models and they checked in these experimental systems the expression and role of MALAT-1. Compared to NP-69 cells, Hep-2 cells, FaDu cells, and CNE-2Z cells showed significantly increased MALAT-1expression. Suppression of MALAT-1 expression significantly inhibited cell proliferation, increased cell population in S phase (*p* < 0.01), decreased cell population in G2/M phase (*p* < 0.01), and attenuated the migration and invasion of thecells [69] (Table 2).

### 5.12. LINC00460 and LINC00520

The expression of LINC00460 in 68 LSCC tissues and paired adjacent normal tissues was recently examined by real-time PCR. The expression of LINC00460 was significantly upregulated in the LSCC tissue compared with that of adjacent normal mucosal tissue (*p* = 0.006). There were no statistical differences of the quantity of LINC00460 expression among supraglottic, glottic and subglottic LSCC (*p* > 0.05). Moreover, LINC00460 had no significant changes in poorly differentiated LSCC when compared with that of well and moderately differentiated LSCC (*p* > 0.05). The expression of LINC00460 in LSCC with lymph node metastasis had no significant changes when compared with that without of lymph node metastasis (*p* > 0.05). Notably, LINC00460 expression in T1+T2 stages patients was significantly lower than T3+T4 stages (*p* < 0.05). Therefore, up-regulation of lncRNA LINC00460 might contribute to the carcinogenesis and development of LSCC, also playing an important biological function [63] (Table 2).

Recently, the expression of LINC00520 in LSCC tissues and paired adjacent normal tissues was determined by real-time PCR. The relationship between the expression of LINC00520 and the clinical and pathological characteristics including clinical stage, pathological type, histological grade and lymph node metastasis of LSCC was analyzed. The LINC00520 expression level was significantly up-regulated in LSCC tissues compared to that of paired adjacent normal tissues (*p* < 0.0001). No statistical differences of the LINC00520 expression levels among supraglottic, glottic and subglottic LSCCs were also found (*p* > 0.05).The LINC00520 expression level had no significant changes in poorly differentiated LSCC compared to well and moderately differentiated counterparts (*p* > 0.05). Moreover, the expression of LINC00520 had no significant difference between T1+T2 stage and T3+T4 stage LSCC tissues (*p* > 0.05). Interestingly, the LINC00520 level in LSCC with lymph node metastasis was significantly higher than that in patients without lymph node metastasis (*p* < 0.01). Therefore, LINC00520 can be considered a radial marker in LSCC [64] (Table 2).

### 5.13. LOC554202

Yang et al. recently reported that LOC554202 expression was higher in LSCC tissues than in the paired adjacent samples. Interestingly, among them, 27 cases (27/40, 67.5%) had higher expression level of LOC554202 in PTC samples compared to the adjacent normal samples. Moreover, the LOC554202 expression was higher in patients with early stage LSCC than in advanced stage LSCC [68]. LOC554202 expression inversely correlated with mi31 expression in LSCC tissues and in order to prove the mechanistic correlation between LOC554202 expression and biological functions in tumours, the LC Hep-2 cells were transfected with cDNA-LOC55420, causing cell growth and increase of percentage population in the S phase of the cell cycle. Opposite effects were caused by the ectopic overexpression of miR31 that was, in turn, down regulated by LOC554202 overexpression. Therefore, a new loop among these two ncRNAs was found that associated LOC554202 with tumorigenic and aggressive phenotype of LSCC (Table 2).

### 5.14. Dleu2

LncRNA Dleu2 and miR-16-1 levels were lower in the laryngeal carcinoma tissue compared to adjacent normal tissues. LncRNA Dleu2 influenced proliferation, migration, and invasion of LC cells through the regulation of miR-16-1. Therefore, lncRNA Dleu2 and miR-16-1 may serve as potential biomarkers and targets for laryngeal cancer diagnosis and treatment [66] (Table 2).

### 5.15. HOXA11-AS

Long noncoding RNA HOXA11 antisense RNA (HOXA11-AS) is involved in tumorigenesis and development of some human cancers. Qu et al. recently reported that, after microarray and qRT-PCR, the levels of HOXA11-AS were significantly higher in LSCC than that in the corresponding adjacent non-neoplastic tissues. ISH revealed that HOXA11-AS was strongly expressed in the nucleus and closely related to the T grade, neck nodal metastasis, and clinical stage. Patients with T3-4 grade, neck nodal metastasis, or advanced clinical stage presented higher HOXA11-AS expression. Kaplan-Meier analysis showed that high HOXA11-AS expression could predict a poor prognosis in LSCC patients. Furthermore, HOXA11-AS knockdown significantly inhibited the growth, migration, and invasion of LSCC cells. Thus, HOXA11-AS plays an oncogenic role in LSCC and may serve as a novel marker and a potential therapeutic target in LSCC patients [62] (Table 2).

### 5.16. CCAT1

The expressions of colon cancer-associated transcript-1 (long non-coding RNA CCAT1) and zinc finger protein, X-linked (ZFX) are higher while microRNA-218 expression is lower in the laryngeal squamous cell cancer tissues than in the normal tissues. MicroRNA-218 overexpression or zinc finger protein, X-linked silencing suppresses proliferation and invasion of laryngeal squamous cancer cells. Knockdown of long non-coding RNA CCAT1 inhibits proliferation and invasion of laryngeal squamous cancer cells, which were reversed by microRNA-218 down-regulation or zinc finger protein, X-linked up-regulation. Moreover, long non-coding RNA CCAT1 silencing inhibits xenograft tumor growth in vivo providing a promising therapeutic target for laryngeal squamous cell cancer patients [59] (Table 2).

### 5.17. RP11-169D4.1

Long non-coding RNA RP11-169D4.1 levels are significantly decreased in LSCC tissues and cell lines, and decreased expression of RP11-169D4.1 indicates a poor prognosis and increases lymph node metastasis in patients with LSCC. RP11-169D4.1 suppresses proliferation and promotes apoptosis in LSCC cells, next RP11-169D4.1 inhibits migration, invasion and epithelial-mesenchymal transitions (EMT) in LSCC cells. RP11-169D4.1 is targeted and inhibited by miR-205-5p.The long non-coding RNA RP11-169D4.1 may serve as a tumor suppressor and a promising therapeutic target in laryngeal cancer, since it could inhibit the process of EMT by regulating CDH1, a well-established tumor suppressor [73] (Table 2).

### 5.18. LINC00261

The expression of long non-coding RNA LINC00261 is decreased in the LSCC tissues compared with the normal tissues. In histological grade grouping, the expression of LINC00261 in T1-T2 stages is significantly higher than T3-T4 stages. The expression of LINC00261 in LSCC with lymph node metastasis is significantly lower than in those without of lymph node metastasis. Down regulation of LINC00261 in LSCC may contribute to carcinogenesis and development of carcinoma [67] (Table 2).

### 5.19. NR027340-SOX2-OT

LncRNAs NR027340, and SOX2-OT and their correlated mRNAs ITGB1, HIF1A, DDIT4 are overexpressed and identified as potential biomarkers in advanced LSCC. This suggests that these up-regulated lncRNAs and mRNAs may represent a new biomarker in diagnosis and prognosis of advanced LSCC, and these targets may be used in potential lncRNA-mediated therapy as well [71,75] (Table 2).

### 5.20. HOTAIR

In LSCC tissues HOTAIR expression, is 16-fold higher compared to normal tissues, and this increase is correlated with advanced tumor grade, lymph node metastasis, poor differentiation and advanced clinical stages. On the other hand, HOTAIR knockdown reduces tumor invasion and increases cell apoptosis in vitro and inhibits LSCC xenograft growth in vivo. HOTAIR serves as an oncogene by inducing hypermethylation of CpG islands in the PTEN gene, reducing the expression of PTEN at mRNA and protein level. Thus, the increased expression of HOTAIR in LSCC promotes cancer progression by activating the phosphatidylinositol 3-kinase (PI3K) signaling pathway [61]. Long non-coding RNA HOTAIR and transmethylase EZH2 play important roles in the progression and development of LSCC. They are over-expressed in LSCC tissue and are related to T phase, pathological grades, and risk of lymphatic metastasis. HOTAIR expression silencing stimulates EZH2 expressing, promotes the cell proliferation, and increased the sensitivity to cis-platinum of the LSCC cells [80] (Table 2).

## 6. Conclusions and Future Perspectives

The understanding of the emerging role of ncRNAs in several cell processes represents a significant advance in many fields of biology. Previous researches have highlighted the dysregulation of ncRNAs in cancer, by providing evidence for potential anticancer targets. Recognized as a genome disease, cancer can be caused by mutations, amplifications, deletions or deregulation of genomic products. Only 2% of the human genome is transcribed and translated into proteins, while up to 70% of it is transcribed into ncRNA. To date, the role of a new class of RNA, with a non-coding function, longer than 200 nucleotides, is of great interest. LncRNAs regulate gene expression by either inducing degradation of the target mRNA or inhibiting its translation. This function is carried out following the pairing of the lncRNA with the regions 3 ’UTRs (untranslated regions) of the target messenger. Based on the target genes, recent studies suggest that lncRNAs are implicated in numerous biological processes such as cell cycle control, apoptosis, development and differentiation, as well as in epigenetic regulation of gene expression [80,81]. The experimental evidence obtained in the last few years show that lncRNAs can represent valid diagnostic and prognostic markers of human tumors. In fact, the aberrant expression of lncRNAs in different tumor types has been correlated with the specific bio-pathological characteristics of the tumor itself, the outcome of the disease and the response to pharmacological treatments. The use of lncRNAs as biomarkers offers many advantages: they are much stable in body fluids (urine, blood, saliva) and can be non-invasively detected by using simple molecular biology techniques (PCR, sequencing) compared to classical biopsies. Moreover, they are differentially expressed in biological fluids and their expression is tissue-specific. On these bases, it could be useful to combine the different lncRNAs with conventional biomarkers in order to obtain an efficient diagnosis and/or prognosis. Some lncRNAs have been already established as sensitive biomarkers in different types of tumors. Recent experimental evidence has shown that lncRNAs such as H19, MALAT1 and HOTAIR are over-expressed in different tumor cell lines [82]. In particular, HOTAIR is over-expressed in primary and metastatic GIST tissues, associated with poor patient survival and the onset of recurrence [83,84]. It is known to be involved in several biological functions that contribute to the characteristics of cell malignancy, which can be attenuated following its removal. MALAT1 lncRNA is a marker of the development of distant metastases and poor prognosis in the early stages of non-small cell lung cancer. Cells with MALAT1 deficiency exhibited severe migration deficits. In a murine model of metastasis MALAT1 knock down significantly reduced tumor nodules in number and size. Also, the alternative inhibition of MALAT1 in cells by means of antisense oligonucleotides caused a drastic reduction of metastasis. MALAT1 could therefore be a marker and an active factor in lung cancer metastasis, as well as a promising target for metastasis prevention therapy [85]. Other studies have led to the identification of several lncRNAs that are overexpressed in a particular subgroup of ovarian cancer, the most aggressive ones. Further analysis revealed that the overexpression of these lncRNAs changes the expression of the proteins that regulate the EMT process [86]. Furthermore, the overexpression of one of the lncRNAs, DNM30S, was significantly correlated with the worsening of survival in patients with ovarian cancer. Most anticancer therapies are not able to permanently heal the tumor and are ineffective in the treatment of patients with advanced forms of carcinoma.

In this context, the lncRNAs, currently little investigated, could provide a valid research field since it is evident that they contribute to the development of carcinoma. The current research aims to analyze the expression of the lncRNAs in cancer patients, in order to identify new prognostic and predictive markers of response to treatment. This also would allow us to shed light on some still little-known molecular mechanisms and identify new therapeutic strategies. Identification of lncRNAs and understanding of their role in HNC and LCs progression yield novel candidates for early detection and metastasis.

## Figures and Tables

**Table 1:** LncRNAs in HNC.

lncRNA	Description	Cancer Type	Expression	Function and Mechanism	Application		Ref
**ADAMTS9-AS2**	-	TSCC	UP	Regulates miR-6010/EZH2 promoting cell migration and invasion via ETM processes	Diagnostic and prognostic biomarker		[37]
**AFAP1-AS1**	LncRNA actin filament-associated protein 1 antisense RNA1	NPC	UP	Affects the expression of cytoskeletally-regulated proteins via inhibiting the Rho/Rac signaling pathway. Promotes cell migration and metastasis	Diagnostic and prognostic biomarker		[38]
**CBR3-AS1**	PlncRNA-1 prostate cancer-up-regulated long noncoding RNA1	ESCC	UP	Promotes cell proliferation and correlates with advanced clinical stage and lymph node metastasis	Diagnostic and prognostic biomarker		[39,40,41]
**ESCCAL-1**	-	ESCC	UP	Knockdown ESCCAL-1 expression increases apoptosis and reduces invasion.	Diagnostic biomarker		[42]
**FIRRE**	LncRNA functional intergenic repeating RNA element	HNSCC	UP	Interacts with the nuclear matrix factor hnRNPU across at least five distinct trans-chromosomal loci	Roles in cell physiology and nuclear architecture		[43,44]
**FOXCUT**	FOXC1 upstream transcript	NPC OSCC	UP	Inhibits cell proliferation and cell migration	Diagnostics biomarker and therapeutics target		[45]
**GAS8-AS1**	LncRNA growth arrest-specific 8-antisense RNA 1	PTC	DOWN	Inhibits cell viability	Diagnostic and therapeutic target		[46]
**H19**	-	NPC HNSCC	UP	Promotes cell invasion by inhibiting the activity of miR-630 and enhancing the expression of zeste homolog2 (EZH2). MiR-675 is correlated with H19.	Prognostic biomarker		[47].
**HNF1A-AS**	Hepatocyte nuclear factor 1A antisense RNA	NPC	UP	Increases mesenchymal proteins N-cadherin and vimentin levels. Reduces the epithelial E-cadherin protein levels. Promotes cell cycle progression, tumor cell proliferation, and migration.	Therapeutics target		[48]
**HOTAIR**	Homeobox transcript antisense RNA	NPC	UP	Promotes cancer growth, angiogenesis and metastasis	Diagnostic and prognostic biomarker		[33,34,35,36]
**LncRNA-LET**	LncRNA-Low Expression in tumor	NPC	DOWN	Associates with cell proliferation, lymph node metastasis. Low levels of LET expression are induced by EZH2-mediated H3K27 histone methylation in Let promoter region	Diagnostic biomarker		[49]
**LncRNA-ROR**	-	NPC	UP	Promotes cell proliferation, metastasis and invasion ability by inducing an EMT phenotype.	Therapeutic target	[50]	
**LOC541471**		HNSCC	UP	Increases lymph node metastasis and perineural invasion	Prognostic factor	[51]	
**MALAT-1**	Metastasis Associated Lung Adenocarcinoma Transcript 1	TSCC	UP	Reduces miR-124 expression and increases growth and metastasis of TSCC cells via targeting jagged 1 (JAG1). Induces EMT, promotes migration ad invasion and inhibits apoptosis of TSCC cells.	Diagnostic and prognostic biomarker	[52]	
**MEG3**	Maternally expressed Gene 3	TSCC	DOWN	Inhibits cell proliferation and the cell cycle, promotes cell apoptosis; DNMT3B is the intermediary by witch miR-26a regulates MEG3 expression.	Diagnostic and prognostic biomarker	[53]	
**PTCSC2**	-	PTC	DOWN	Influences genes expression and cell cycle	-	[54]	
**PTCSC3**	-	PTC	DOWN	Inhibits cell grow, influences the expression of genes involved in DNA replication, recombination and repair; cellular movement; tumor morphology and cell death.	Diagnostic biomarker	[55]	
**PVT1**	-	TC	UP	Promotes cell proliferation and cell cycle progression in TC by recruiting EZH2 and regulating TSHR	Diagnostic biomarker	[56]	
**UCA1**	Urothelial carcinoma- associated 1	TSCC	UP	Promotes migration	Diagnostic and therapeutic strategy	[57]	

**Table 2:** LncRNAs in LSCC.

lncRNA	Description	Cancer Type	Expression	Function and Mechanism	Application	Ref
**CCAT1**	Colon cancer-associated transcript-1	LSCC	UP	Activates cancer cell proliferation, migration and invasion	Diagnostic and prognostic biomarker	[59]
**DGCR5**	LncRNA Di George syndromecriticalregion gene5	LSCC	UP	Induces cancer stem cells-like properties by sponging miR-506 trough Wnt signaling activation	Diagnostic biomarker	[58]
**H19**	-	LSCC	UP	Increases DNA methylation by repressing miR-148a-3p	Prognostic biomarker	[60]
**HOTAIR**	Homeobox transcript antisense RNA	LSCC	UP	Promotes cancer growth, angiogenesis and metastasis	Diagnostic and prognostic biomarker	[61]
**HOXA11-AS**	HOXA11 antisense RNA	LSCC	UP	Promotes cancer growth, angiogenesis and neck nodal metastasis	Diagnostic biomarker and therapeutic target	[62]
**LINC00460**	Long intergenic non-protein coding RNA 460	LSCC	UP	Contributes to the carcinogenesis and development of LSCC	-	[63]
**LINC00520**	Long intergenic non-protein coding RNA 520	LSCC	UP	Induces metastasis. Promotes cell growth, cell cycle and cell invasion. Suppresses miR-31 expression and promotes RhoA expression.	Diagnostic and prognostic biomarker	[64]
**LIN00668**	Long intergenic non-proteincoding RNA 668	LSCC	UP	Promotes cell proliferation, migration and invasion. Associates with age, pathological differentiation degree, T stage and Lymph node metastasis.	Diagnostic and prognostic biomarker	[65]
**LncRNA Dleu2**	-	LSCC	DOWN	Influences the proliferation, migration, and invasion of laryngeal cancer cells through miR-16-1	Diagnostic biomarker and therapeutic target	[66]
**LIN00261**	-	LSCC	DOWN	Contributes to the carcinogenesis and development of LSCC	Therapeutic target	[67]
**LOC554202**	-	LSCC	UP	Promotes cell growth, cell cycle and invasion; suppresses miR-31 expression and promotes RhoA expression	-	[68]
**MALAT-1**	Metastasis Associated Lung Adenocarcinoma Transcript 1	LSCC	UP	Promotes cell proliferation and inhibits apoptosis	Diagnostic and prognostic biomarker	[69]
**NEAT1**	Nuclear enrich abundant transcript 1	LSCC	UP	Promotes tumor growth and cell cycle progression in LSCC by regulating miR-107/CD46 pathway	-	[70]
**NR027340**	-	LSCC	UP	-	Diagnostic biomarker and target therapy	[71]
**PCAT 19**	-	LSCC	UP	Decreases cells proliferation, inhibits glycolysis by modulating the mir-182/pdk4 axis	Diagnostic biomarker	[72]
**RP11-169 D4.1-001**	-	LSCC	DOWN	Increases lymph node metastasis in patients. Suppresses proliferation and promote apoptosis. Inhibits migration, invasion and epithelial-mesenchymal transitions (EMT)	Diagnostic and prognostic biomarker	[73]
**ST7-AS1**	LncRNA suppressor of tumorigenicity 7 antisense RNA 1	LCSS	UP	Promotes migration. Interacts with CARMI (coactivator-associated arginine methyltransferase) protecting it from ubiquitin-dependent degradation	Diagnostic biomarker	[74]
**SOX2-OT**	-	LSCC	UP	-	Diagnostic and prognosticbiomarker	[75]
**SNAR**	LncRNA small NF90-associated RNA	LCSS	UP	Correlates with cell proliferation, migration and invasion. Its action is related to TGF-ß1	-	[76]
**SNHG1**	LncRNA small nucleolar RNA host gene 1	LSCC	UP	Promotes cell proliferation, migration and invasion. Moreover, induces cell apoptosis and promotes YAPI expression and Hippo signaling activity by miR-375	Diagnostic biomarker	[77]
**TUG1**	LncRNA taurine upregulated gene1	LSCC	UP	Associates with lymph node metastasis. TUG1 silencing inhibits proliferation, cell-cycle progression, migration and invasion	Prognostic biomarker	[78]
**UCA1**	Urothelial carcinoma- associated 1	LSCC	UP	Promotes proliferation, migration and invasion by activating Wnt/β-catenin signaling pathway	Diagnostic biomarker	[79]

## Abbreviations

HNC	head and neck cancer
HNSCC	head and neck squamous cell carcinoma
HPV	human papillomavirus
LncRNAs	long non-coding RNAs
OSCC	oral squamous cell carcinoma
LSCC	laryngeal squamous cell carcinoma
TSCC	tongue squamous cell carcinoma
HSCC	hypopharyngeal squamous cell carcinoma
NPC	nasopharyngeal carcinoma
TC	thyroid cancer
PTC	papillary thyroid cancer
